# Alcohol use and internal migration in Nepal: a cross-sectional study

**DOI:** 10.1136/jech-2022-220030

**Published:** 2023-07-04

**Authors:** Dirgha J. Ghimire, Faith Cole, Sabrina Hermosilla, William G. Axinn, Corina Benjet

**Affiliations:** 1 Institute for Social Research, University of Michigan, Ann Arbor, MI, U.S.A; 2 Epidemiology and Psychosocial Research, National Institute of Psychiatry, Mexico City, Mexico

**Keywords:** Alcohol use, Alcohol use disorder (AUD), internal migration, LMICs, South Asia

## Abstract

**Background:**

Alcohol use is a leading cause of disease. Although low-and-middle-income countries (LMICs) have lower per capita alcohol consumption, the alcohol-attributable disease burden is high in these settings with consumption increasing. LMICs are also experiencing unprecedented levels of internal migration, potentially increasing mental stress, changing social restrictions on drinking, and increasing alcohol availability. We assessed the relationship between internal migration, opportunity to drink, and the transition from first use to regular alcohol use and alcohol use disorders (AUD) in Nepal, a low-income, South Asian country.

**Methods:**

A representative sample of 7,435 individuals, aged 15–59 from Nepal were interviewed in 2016–2018 (93% response rate) with clinically validated measures of alcohol use and disorders and life history calendar measures of lifetime migration experiences. Discrete-time hazard models assessed associations between migration and alcohol use outcomes.

**Results:**

Net of individual sociodemographic characteristics, internal migration was associated with increased odds of opportunity to drink (OR=1.32, 95% CI=1.14–1.53), onset of regular alcohol use given lifetime use (OR=1.29, 95% CI=1.13–1.48), and AUD given lifetime use (OR=1.24, 95% CI=0.99–1.57). The statistically significant association between internal migration and opportunity to drink was specific to females, whereas the associations between migration and regular use and disorder were statistically significant for males.

**Conclusions:**

Despite high rates of internal migration worldwide, most research studying migration and alcohol use focuses on international migrants. Findings suggest that internal migrants are at increased risk to transition into alcohol use and disorders. Support services for internal migrants could prevent problematic alcohol use among this underserved population.

## Introduction

Alcohol use disorder (AUD) is a driving cause of premature death and disability globally [1]. Alcohol consumption is associated with over 60 diseases [1, 2] and it is estimated that over 100 million people live with alcohol dependence worldwide [1]. Alcohol use-associated mortality is higher than that caused by tuberculosis, HIV/AIDs, and diabetes [3]. The distribution of the alcohol-attributable disease burden is larger in low-and-middle-income countries (LMICs) than high-income countries [3, 4]. Historically, LMICs reported lower per-capital alcohol use compared to high-income countries [5], but a higher prevalence of hazardous drinking patterns among those who drink [6]. Per capita alcohol consumption and heavy episodic drinking are highly gendered (males are at higher risk for both) [6, 7] and increasing in LMICs [4, 5, 8], particularly in Southeast Asia [3]. Per capita alcohol consumption and the burden of alcohol-attributable disease are expected to grow rapidly in LMICs [5, 6, 9].

LMICs are experiencing increasing internal migration, particularly from rural to urban areas [10]. Although international migration is primarily dominated by men and is temporary, internal migrants are often women and young adults moving from rural-to-urban areas, globally [11] and can be temporary or permanent [12, 13].

Migration may affect alcohol use behaviors through multiple pathways. First, migration likely changes one’s access to alcohol. Those migrating for work may have greater disposable income to spend on alcohol, and migration from rural-to-urban settings may increase access to commercially-produced alcohol [11]. Second, migration away from family and friends may lessen the influence of social restrictions on drinking [6, 11]. Nepal is a predominantly Hindu society historically exposed to high levels of religious prohibitions against alcohol consumption. With reduced social restrictions, internal migrants’ daily social lives become less formal and more poorly regulated than in their place of origin. As women are more likely to adhere to these restrictions than men, and drinking alcohol is viewed as inappropriate/immoral for women, leading to stigmatization, this difference is easily observed across all processes leading to AUD [12, 14]. This substantial gender difference means internal migration is more likely to give women new access to alcohol they did not have before moving than men. Finally, migration, which often involves separation from family, exposure to an unknown setting, and discrimination or taxing work conditions, can be stressful, potentially leading to increased and heavy episodic drinking. Thus, mechanisms that contribute to the association between migration and drinking might differ by gender because the reasons for migration and social restrictions vary by gender.

Studies have investigated associations between international migration from low-income settings to high-income settings and alcohol use [15–17], often finding that international migrants report lower burdens of alcohol use and disorder compared to native-born populations [18–21]. However, research focused on internal migration within LMIC varies across settings [16]. Although one study suggests there is no difference in alcohol use and AUD between migrants and non-migrants [22], research largely suggests that internal migration is associated with increased at-risk drinking and poor health [17, 21, 23, 24]. These studies include migrants moving from rural areas to mega cities, whereas internal Nepali migrants move between rural areas or to small town/urban areas. However, most migration research focuses on the prevalence of AUD or at-risk drinking among migrants and largely overlooks the processes leading to AUD among internal migrants, an important intervention point.

We investigate the relationship between internal migration and transitions into alcohol use stages in the Chitwan Valley Family Study (CVFS), a mixed-method panel study in Nepal that follows all migrants. CVFS features detailed migration histories linked to clinically-validated alcohol use outcomes [12, 25] measured with a life history calendar (LHC) that improves retrospective reporting [26].

Previous research in this sample found that males were at increased risk for all stages of alcohol use compared to females; ethnic groups with a history of alcohol restrictions were associated with lower risk of early alcohol use stages, but not AUD; and younger birth cohorts were associated with increased risk of all alcohol use stages [12]. Although little is known about how regional mobility affects alcohol use patterns [7, 20, 23], we hypothesize that individuals with a history of internal migration will be at increased risk for the onset of opportunity to drink, regular alcohol use, and AUD compared to those without a history of migration.

## Methods

### Design

Western Chitwan is an ethnically-diverse primarily rural agricultural, migrant-sending area in south-central Nepal. Nepal has a long history of population mobility. Most migrants (60%) are internal migrants moving from rural-to-urban settings [27–29], despite high levels of international migration [15, 30]. Nepal is projected to have one of the fastest rates of urbanization globally [27]. Internal migrants are largely female, younger, and somewhat more educated than international migrants [30]. Reasons for internal migration are highly gendered: females move primarily for marriage (58.9%), family reasons (20.1%), study (14.4%), and work (6.0%), males move primarily for work (49.7%) and study (32.0%). Most internal migrants are employed in agriculture before migrating, and most are employed in service industries at their destination [13, 31].

The CVFS is a multilevel, mixed-method panel study of communities, households, and individuals. Launched in 1995, CVFS selected a systematic probability sample of 151 neighborhoods (5–15 households) in Western Chitwan. These neighborhoods were selected as an equal-probability, systematic sample of neighborhoods, and the characteristics in this sample closely resemble the demographic characteristics of Western Chitwan. All participants, including migrants irrespective of destinations, were followed with sample refreshes maintaining the representativeness of Western Chitwan. CVFS methods of frequent re-contact and mixed-mode data collection generated high-quality data with greater than 90% response rates and low item missing data [32, 33].

All residents aged 15–59 living in one of the 151 neighborhoods were eligible to participate. In 2016–18, the CVFS measured lifetime prevalence and onset of alcohol use and disorders and migration history among all participants [12]. Our analytic sample is sourced from the 2018 general population, with some restrictions. To avoid confounding in-migration with out-migration, we restricted our sample to person-years after the respondent moved to a CVFS neighborhood. This results in a sample of 7,435 individuals who lived in a CVFS neighborhood before the onset of their first alcohol use stage. This sample is slightly younger and more female compared to the general sample (for the general sample characteristics see [12, 14]).

### Measures

#### Alcohol use and disorders

Opportunity to drink, regular alcohol use, and AUD were measured with the Composite International Diagnostic Interview (CIDI) [34, 35]. The CIDI instrument was carefully translated and adapted to create the Nepal-specific CIDI [36]. The instrument was paired with an LHC to improve recall of the timing of alcohol use and symptom onset. Rigorously trained, professional interviewers administered the Nepal CIDI using computer-assisted personal interviewing.

First opportunity to drink was evaluated by the question “About how old were you the very first time you had an opportunity to drink alcohol?”, with opportunity to drink explained to the respondent as “anytime someone either offered you alcohol or you were present when others were drinking and could have drunk if you wanted to.” Descriptions of alcoholic beverages included local alcoholic beverages (*jad and rakshy*), beer, wine, and hard liquor. Regular alcohol use was measured through the question, “How old were you when you first started drinking at least 12 drinks in a year?” Age of onset for opportunity to drink and regular alcohol use were constructed using the age the respondent reported for these questions. Lifetime alcohol use measures were coded 1 if the respondent reported age of onset of that alcohol use stage, and 0 for “Never.” Finally, lifetime AUD was evaluated according to DSM-IV diagnostic criteria for alcohol abuse (ALA) and alcohol dependence (ALD). We combined ALA and ALD into AUD, defined for the dichotomous measure as any lifetime ALA or ALD and the age of onset as the age at which the respondent first met AUD criteria [25, 37].

#### Internal migration

The LHC also measured respondents’ lifetime migration histories and recorded their annual place of residence from birth through the interview date. Using LHC migration information, we created a time-varying measure, coded 1 if the respondent lived away from home within Nepal in the previous year and 0 otherwise. International migration is also likely to alter alcohol use behaviors, but estimating the association between international migration and alcohol use is complicated by the heterogeneity in migration destinations that range from India to Western Europe and North America. Moreover, the small number of migrants in each destination country substantially limits estimation power. Because international migration may also have long-term consequences even after returning to place of origin, we excluded person-years after the first time an individual migrated internationally from our analyses.

#### Sociodemographic measures

We included sociodemographic covariates associated with alcohol use in Nepal: *gender*, *age, birth cohort* (1957–1971, 1972–1981, 1982–1991, 1992–2001), *ethnicity,* and *education* [12]. Ethnicity was coded into the five most common ethnic groups in Western Chitwan: Brahmin/Chhetri, Hill Janajati, Newar, Terai Janajati, or Dalit [38]. Educational attainment was classified as achieving a “School Leaving Certificate” (SLC). The SLC is earned by scoring highly on a nationally standardized exam offered after the completion of tenth grade, and variance in this attainment reflects recent changes across cohorts in access to schools. Because proximity to an urban center is likely to be associated with the availability of commercial alcohol and social organization of daily social life, we also included a covariate indicating the distance to an urban center from the respondent’s residence before the internal migration.

### Analytical Approach

We used event history methods to model the risk of the onset of alcohol use stages. Because data are precise to the year, we used discrete-time methods [39]. This requires an annual record of an onset of a specific alcohol use stage (experienced an onset or not in a specific year (1,0)), labeled as person-exposure to a specific alcohol use stage for which person-year is the unit of analysis. The outcome for each model was the first onset of the focal alcohol use stage, which included first opportunity to drink, first regular alcohol use given lifetime alcohol use, and first AUD given lifetime use. We considered individuals to be at risk of the onset of alcohol use stages starting at age 10 or the first year they lived in a CVFS neighborhood. This excluded individuals from the model if their onset of the focal alcohol use stage was before they lived in a CVFS neighborhood. To estimate the discrete-time hazard models, we used logistic regression, with one-tailed test. We transformed the raw coefficients by exponentiating them; the coefficients are estimates of the multiplicative effects on the odds of the onset of the alcohol use stage in any one-year interval. Our time-varying measure of internal migration was measured in the year *before* the current year of onset of alcohol use stage. Because individuals are clustered within neighborhoods, we adjusted models for neighborhood-level clustering.

## Results

Table 1 presents sample characteristics: 33.01% migrated internally, 58.01% were female, 40.70% passed the SLC, 45.70% were Brahmin/Chhetri, 20.09% Terai Janajati, 16.79% Hill Janajati, 12.17% Dalit, and 5.25% Newar. Our sample included slightly larger younger cohorts compared to the full sample, with 37.53% born between 1992–2004; 28.14% between 1982–1991, 17.98% between 1972–1981, and 16.36% born before 1972. The mean age was 31.60 years (SD=11.92) with a range of 15–63 years. Regarding alcohol use stages, 60.90% ever had an opportunity to drink, 27.46% ever had regular alcohol use, and 5.07% ever met diagnostic criteria for an AUD. The prevalence of each alcohol use stage using the full sample of 10,714 individuals is published elsewhere [12].

Table 2 presents estimates of the association between internal migration in the prior year and the onset of alcohol use stages. Model 1 shows the association between internal migration and first opportunity to drink, controlling for gender, ethnicity, birth cohort, education, distance to an urban center, age, and age squared. Internal migration in the prior year was associated with increased odds of opportunity to drink (OR=1.32, CI=1.14–1.53, p<0.001).

Model 2 estimated the association between internal migration in the prior year and onset of regular use, given lifetime use. We controlled for the same sociodemographic covariates. Internal migration was associated with greater odds of the onset of regular alcohol use (OR=1.29, CI=1.13–1.48, p<0.001) among those who ever had a drink, compared to those who did not migrate internally in the previous year.

Finally, Model 3 estimates the association between internal migration in the prior year and the onset of AUD, conditional on lifetime use and controlling for sociodemographic covariates. Internal migration was associated with greater odds of onset of AUD (OR=1.24, CI=0.99–1.57, p<0.05) among those who ever had a drink, compared to those who did not migrate in the prior year.

Next, because the probability of migration and alcohol use are likely to vary by gender, we estimated models of alcohol use for females and males separately. Tables 3 and 4 present the odds of opportunity to drink, regular use, and AUD by gender. Internal migration was associated with increased odds of opportunity to drink for females (OR=1.36, CI=1.19–1.55, p<0.001), but not for males (OR=1.19, CI=0.69–2.03, p=0.53). Yet, internal migration was associated with increased odds of both regular use (OR=1.29, CI=1.00–1.65, p<0.05) and AUD (OR=1.25, CI=0.99–1.59, p<0.05) for men, but not for women. Since a small number of women had regular alcohol use and AUD, the non-significant results for women could be an artifact of sample size and therefore should be interpreted cautiously.

Furthermore, alcohol use is likely associated with an individual’s age or birth year. We estimated models of alcohol use opportunity, regular use, and AUD for four birth cohorts: born before 1972; 1972–1981; 1982–1991; and 1992–2004. Net of other socioeconomic factors, internal migration was associated with increased odds of alcohol use outcomes for most cohorts (significant in eight of twelve models).

Finally, we estimated all models with two other measures of internal migration: i) number of internal migration periods, and ii) number of years of internal migration. The results (available upon request) of these analyses closely resembled the results presented above, providing additional confidence in these findings.

## Discussion

We investigated the relationship between internal migration and alcohol use in a low-income country. Results show internal migration was associated with increased odds of all three stages of alcohol use: opportunity to drink, regular use given lifetime use, and the onset of AUD given lifetime use. In contrast to the literature investigating alcohol use among international migrants [18–21], our findings suggest that internal migrants are at increased risk for alcohol use and developing an AUD. This provides insight into the processes leading to AUD in Nepal, where internal migration is common and likely increasing, and increased imports and industrial production are making alcohol more accessible [40].

Additionally, these associations vary by gender, given that both alcohol use patterns and migration rates and reasons for migration are highly gendered in Nepal. Internal migration was associated with increased odds of opportunity to drink for women, but not for men. However, internal migration was associated with increased odds of regular use and AUD for men. This suggests that in settings with social restrictions against drinking, which are especially strict for Nepali women given social taboos, migration may increase women’s opportunity to drink. Notably, we did not find a significant association between women’s migration and regular alcohol use, suggesting that even if migration increases women’s opportunity to drink, it may not push them to drink regularly. Male migrants did not have increased odds of the opportunity to drink. This could be because Nepali men face looser restrictions against alcohol use and tend to have an earlier onset of alcohol use, potentially resulting in the opportunity to drink before migrating. Yet, men had increased odds of initiating regular alcohol use and developing an AUD in the year after migrating, suggesting their increased risk is due to other mechanisms, such as stress or increased resources to purchase alcohol. These results are contrary to the healthy migrant effects documented in international migrant studies that show international migrants to the U.S. or Europe are less likely to report hazardous drinking or be diagnosed with recent and lifetime substance use disorders compared to native-born residents [18–21], this difference could be due to the preexisting high level of drinking culture in U.S. or European societies.

These findings have some limitations. First, our measures of AUD and migration come from a retrospective survey, thus recall bias may lead to underreporting of alcohol use, internal migration, and inaccuracies in the ages of events. Counter to other retrospective migration studies, we minimized this bias by using the LHC method, effective in providing memory cues and improving retrospective measurement [26]. Second, the historical social prohibition of alcohol use in certain ethnic groups, particularly Brahmin/Chhetri, may have led to underreporting of alcohol use. Third, because of the heterogeneous nature of the international destinations and small migrant numbers in several countries, this study focused on internal migration only. Fourth, because very few women had AUD within our sample, our estimates of associations between migration and AUD for women should be interpreted with caution. Of these limitations, the only limitation that might bias our results is the plausible underreporting by Brahmin/Chhetri ethnic groups or females, which might lead to more conservative results or a tendency toward null findings for those groups.

Despite these limitations, our study has several strengths. This is the first study to investigate alcohol use transitions among internal migrants, demonstrating the processes associated with alcohol use and disorders among migrants. Second, we use a large population-representative sample from a region with social restrictions against drinking, allowing us to study the relationship between adult migration and the opportunity to drink. Third, the study setting is similar to other South Asian settings in both migration and alcohol use patterns. This provides insights into the relationship between internal migration and alcohol use outcomes that are relevant to the most populated and rapidly growing region of the world.

## Conclusion

This study provides insights that can guide social support services for new, internal migrants in settings characterized by urbanizing populations, increasing alcohol availability, and historical restrictions against alcohol use. Findings that internal migration in the prior year increased the odds of opportunity to drink, regular alcohol use given lifetime use, and AUD given lifetime use suggest that programs aimed at preventing risky alcohol use behaviors may benefit from providing resources for new migrants. This is an important contribution, since most research studying the social support services available to migrants focuses on those for international migrants. Although we observed an association between internal migration and increased odds of opportunity to drink for women, we did not observe an association between internal migration and later alcohol use stages for women, and men were at elevated risk of transitions to regular use and AUD. Although internal migration may increase the accessibility of alcohol for women who have not had the opportunity to drink, interventions aimed at preventing risky alcohol use behaviors may want to focus resources on preventing transitions from first alcohol use to later alcohol use stages in men.

## Figures and Tables

**Table 1:** **Descriptive characteristics of the analytic sample** (N=7,435)^a^

	Male (N=3,122)		Female (N=4,313)		Overall (N=7,435)	
	N	Percent	N	Percent	N	Percent
**Lifetime Migration Experience** ^b^						
Never migrated	786	25.18	2,437	56.50	3,223	43.35
Ever migrated internally	906	29.02	1,536	35.61	2,442	32.84
Ever migrated internationally	1,430	45.80	340	7.88	1,770	23.81
**Lifetime Opportunity to Drink**						
No	245	7.85	2,662	61.72	2,907	39.10
Yes	2,877	92.15	1,651	38.28	4,528	60.90
**Lifetime Regular Alcohol Use**						
No	1,214	38.89	4,179	96.83	5,393	72.54
Yes	1,908	61.11	134	3.11	2,042	27.46
**Lifetime AUD** ^c^						
No	2,761	88.44	4,297	99.63	7,058	94.93
Yes	361	11.56	16	0.37	377	5.07
**Birth Cohort**						
1992–2004	1,262	40.42	1,528	35.43	2,790	37.53
1982–1991	833	26.68	1,259	29.19	2,092	28.14
1972–1981	553	17.71	784	18.18	1,337	17.98
before 1972	474	15.18	742	17.20	1,216	16.36
**Ethnicity**						
Brahmin/Chhetri	1,384	44.33	2,014	46.70	3,398	45.70
Hill Janajati	509	16.30	739	17.13	1,248	16.79
Dalit	412	13.20	493	11.43	905	12.17
Newar	167	5.35	223	5.17	390	5.25
Terai Janajati	650	20.82	844	19.57	1,494	20.09
**Level of education**						
SLC or more^d^	1,449	46.41	1,580	36.63	3,029	40.7
No SLC	1,673	53.59	2,733	63.37	4,406	59.26
					**N**	**Mean**
**Distance to urban center (kilometers)**					7435	13.76

a) The analytic sample includes individuals who were interviewed in the 2016–2018 CIDI interview and who lived in a CVFS neighborhood prior to the onset of their first alcohol use stage.

b) As individuals can be both internal and international migrants, for the descriptive characteristics we counted individuals who ever migrated internationally as international migrants, and individuals who migrated within Nepal, but never outside Nepal as internal migrants. In the hazard models, individuals are no longer considered at risk for internal migration after the first time they ever migrate internationally.

c) AUD refers to any lifetime AUD, including abuse (ALA) or dependence (ALD).

d) SLC=School Leaving Certificate.

**Table 2:** Multi-level hazard model estimates of the association between individual migration and transitions in alcohol use stages^a,b,c^

	Model 1: Opportunity to drink		Model 2: Regular use given lifetime use		Model 3: AUD given lifetime use	
	Odds ratio	95% Confidence interval	Odds ratio	95% Confidence interval	Odds ratio	95% Confidence interval
**Migration**						
Internal migration (prior year)	1.32***	(1.14–1.53)	1.29***	(1.13–1.48)	1.24*	(0.99–1.57)
**Gender** (ref: Female)						
Male	6.08***	(5.31– 6.96)	5.04***	(4.09–6.20)	3.60***	(2.37–5.46)
**Ethnicity** (ref: Brahmin/Chhetri)						
Newar	1.38**	(1.08–1.77)	1.17	(0.91–1.51)	0.90	(0.54–1.50)
Dalit	1.47***	(1.22– 1.77)	1.25*	(1.03–1.51)	1.72***	(1.22–2.42)
Hill Janajati	1.99***	(1.70–2.34)	1.64***	(1.38–1.96)	1.74***	(1.27–2.38)
Terai Janajati	1.74***	(1.49–2.03)	1.38***	(1.16–1.63)	1.13	(0.80–1.59)
**Birth cohort** (ref: before 1972)						
1972–1981	1.34**	(1.10–1.64)	1.16*	(0.97–1.38)	1.25	(0.90–1.76)
1982–1991	2.36***	(1.94–2.87)	1.97***	(1.65–2.35)	3.77***	(2.67–5.34)
1992–2004	5.69***	(4.61–7.03)	4.51***	(3.72–5.46)	8.27***	(5.43–12.53)
**Level of education** (ref: No SLC)^d^						
SLC or more	1.0	(0.91–1.17)	0.66***	(0.58–0.75)	0.74*	(0.58–0.95)
**Distance to urban center (kilometers)**	0.99	(0.99–1.02)	0.96*	(0.97–1.00)	0.99	(0.97–1.03)
**Age** ^e^	1.61***	(1.54–1.69)	1.68***	(1.60–1.76)	1.41***	(1.33–1.51)
**Age squared** ^c^	0.99***	(0.99–0.99)	0.99***	(0.99–0.99)	1.00***	(0.99–1.00)
Person-year	79,539		28,453		47,307	
Individuals	7,435		2,710		3,468	
Deviance	27,750.8		11,263.1		3,492.5	
−2 Res log likelihood	600,573.3		181,951.3		386,706.3	

a) Exponentiated coefficients; 95% confidence intervals in parentheses

* p<.05

** p<.01

*** p<.001

all probabilities are one-tailed.

b) All models were estimated using multivariate logistic regression. Individuals start contributing person-years at age 10 or when they move to a CVFS neighborhood, then contribute one person-year each year until the onset of the focal alcohol use stage, first international migration, or the CIDI interview. All models were adjusted for neighborhood clustering.

c) Individuals who met diagnostic criteria for the focal alcohol use stage prior to age 10 or prior to moving to a CVFS neighborhood were excluded from the models. Person-years with missing data on any covariate were excluded from the models.

d) SLC=School Leaving Certificate.

e) Age and age squared are time-varying. Age squared is an indicator of the decaying effect of age.

**Table 3:** Multi-level hazard model estimates of the association between female internal migration and transitions in alcohol use stages^a,b,c^

	Model 1: Opportunity to drink		Model 2: Regular use given lifetime use		Model 3: AUD given lifetime use	
	Odds ratio	95% Confidence interval	Odds ratio	95% Confidence interval	Odds ratio	95% Confidence interval
**Migration**						
Internal migration (prior year)	1.36***	(1.19–1.55)	0.88	(0.53–1.46)	0.71	(0.27–1.87)
**Ethnicity (**ref: Brahmin/Chhetri)						
Newar	1.36**	(1.06–1.73)	0.30*	(0.10–0.87)	0.00	(0.00–0.00)
Dalit	1.60***	(1.32–1.93)	1.94*	(0.93–4.04)	8.19**	(1.44–46.65)
Hill Janajati	2.50***	(2.13–2.93)	1.31	(0.67–2.57)	2.55	(0.43–15.22)
Terai Janajati	2.01***	(1.69–2.39)	1.30	(0.67–2.54)	2.24	(0.41–12.25)
**Birth cohort** (ref: before 1972)						
1972–1981	1.38***	(1.13–1.67)	1.54*	(1.01–2.35)	1.24	(0.54–2.89)
1982–1991	2.60***	(2.13–3.18)	2.00**	(1.14–3.49)	1.84	(0.49–6.92)
1992–2004	6.83***	(5.43–8.58)	6.46***	(3.18–13.13)	52.77***	(11.82–235.47)
**Level of education** (ref: No SLC)^d^						
SLC or more	1.20**	(1.06–1.35)	0.35**	(0.18–0.69)	0.06**	(0.01–0.39)
**Distance to urban center (kilometers)**	1.00	(0.98–1.02)	0.94	(0.91–1.02)	1.01	(0.89–1.21)
**Age** ^e^	1.27***	(1.23–1.31)	1.29***	(1.17–1.43)	1.59***	(1.28–1.99)
**Age squared** ^e^	1.00***	(1.00–1.00)	1.00***	(1.00–1.00)	0.99***	(0.99–1.00)
Person-year	53,129		4,989		7,332	
Individuals	4,312		359		432	
Deviance	12,951.8		1,177.5		3,492.5	
−2 Res log likelihood	356,667		32,718		74,777	
						
						

a) Exponentiated coefficients; 95% confidence intervals in parentheses

* p<.05

** p<.01

*** p<.001

all probabilities are one-tailed.

b) All models were estimated using multivariate logistic regression. Individuals start contributing person-years at age 10 or when they move to a CVFS neighborhood, then contribute one person-year each year until the onset of the focal alcohol use stage, first international migration, or the CIDI interview. All models were adjusted for neighborhood clustering.

c) Individuals who met diagnostic criteria for the focal alcohol use stage prior to age 10 or prior to moving to a CVFS neighborhood were excluded from the models. Person-years with missing data on any covariate were excluded from the models.

d) SLC=School Leaving Certificate.

e) Age and age squared are time-varying. Age squared is an indicator of the decaying effect of age.

**Table 4:** Multi-level hazard model estimates of the association between male internal migration and transitions in alcohol use stages^a,b,c^

	Model 1: Opportunity to drink		Model 2: Regular use given lifetime use		Model 3: AUD given lifetime use	
	Odds ratio	95% Confidence interval	Odds ratio	95% Confidence interval	Odds ratio	95% Confidence interval
**Migration**						
Internal migration (prior year)	1.19	(0.69–2.03)	1.29*	(1.00–1.65)	1.25*	(0.99–1.59)
**Ethnicity (**ref: Brahmin/Chhetri)						
Newar	1.31	(0.53–3.21)	1.24	(0.79–1.95)	0.95	(0.57–1.58)
Dalit	1.36	(0.70–2.63)	1.20	(0.85–1.70)	1.59**	(1.12–2.27)
Hill Janajati	1.58+	(0.87–2.88)	1.69***	(1.24–2.30)	1.81***	(1.32–2.48)
Terai Janajati	1.43	(0.81–2.52)	1.34*	(1.00–1.79)	1.14	(0.80–1.63)
**Birth cohort** (ref: before 1972)						
1972–1981	1.49	(0.72–3.05)	1.15	(0.82–1.60)	1.22	(0.86–1.74)
1982–1991	2.86**	(1.42–5.73)	1.93***	(1.39–2.68)	3.82***	(2.67–5.47)
1992–2004	6.29***	(3.05–12.96)	4.42***	(3.11–6.29)	7.86***	(5.12–12.05)
**Level of education** (ref: No SLC)^d^						
SLC or more	0.82	(0.52–1.27)	0.66***	(0.53–0.84)	0.75*	(0.58–0.97)
**Distance to urban center (kilometers)**	1.00	(0.95–1.06)	0.97	(0.96–1.01)	0.99	(0.97–1.03)
**Age** ^e^	2.22***	(1.73–2.84)	1.92***	(1.72–2.14)	1.42***	(1.33–1.51)
**Age squared** ^e^	0.99***	(0.98–0.99)	0.99***	(0.99–0.99)	1.00***	(0.99–1.00)
Person-year	26,410		23,464		39,975	
Individuals	3,123		2,351		3,036	
Deviance	14,079.6		9,953.4		3,215.5	
−2 Res log likelihood	236,334		176,137		321,541	
						
						

a) Exponentiated coefficients; 95% confidence intervals in parentheses

* p<.05

** p<.01

*** p<.001

all probabilities are one-tailed.

b) All models were estimated using multivariate logistic regression. Individuals start contributing person-years at age 10 or when they move to a CVFS neighborhood, then contribute one person-year each year until the onset of the focal alcohol use stage, first international migration, or the CIDI interview. All models were adjusted for neighborhood clustering.

c) Individuals who met diagnostic criteria for the focal alcohol use stage prior to age 10 or prior to moving to a CVFS neighborhood were excluded from the models. Person-years with missing data on any covariate were excluded from the models.

d) SLC=School Leaving Certificate.

e) Age and age squared are time-varying. Age squared is an indicator of the decaying effect of age.

## Data Availability

The data used in this study come from the Chitwan Valley Family Study (CVFS). De-identified CVFS data and documentation are archived at the Inter-university Consortium for Political and Social Research (ICPSR). For more information on using CVFS data, please go to https://cvfs.isr.umich.edu/. The mental health data presented here are available upon request and will be archived at ICPSR soon. They will be available to the scientific community following ICPSR protocols for restricted-use data.

## Key Elements

### What is already known about this topic

- Research on internal migration and alcohol use within LMICs varies, with some studies suggesting few or no differences in alcohol use and AUD between migrants and non-migrants, and others suggesting that internal migration is associated with increased at-risk drinking and poor health.
- Most migrant research focuses on the prevalence of AUD or at-risk drinking; little research has investigated the processes associated with transitioning to AUD among internal migrants.

### What this study adds

- This study demonstrates that internal migration in the previous year increased the odds of opportunity to drink, regular alcohol use given lifetime use, and AUD given lifetime use.
- The increased odds of transitioning from ever use to regular use and AUD is only associated with men, not with women.

### How this study might affect research, practice or policy

- Only male internal migrants were at elevated risk of transitions to regular use and AUD, so interventions aimed at preventing alcohol use behaviors may benefit from focusing on preventing transitions from first alcohol use to later alcohol use stages in male internal migrants.
